# In Reply: Estimating Appendicular Skeletal Muscle Mass in Studies Based on the CHARLS Database

**DOI:** 10.1002/jcsm.70039

**Published:** 2025-07-26

**Authors:** Ya‐Xi Luo, Xiu‐Qing Yao

**Affiliations:** ^1^ Department of Rehabilitation The Second Affiliated Hospital of Chongqing Medical University Chongqing China

We thank Liu et al. for their commentary on our recent study exploring the bidirectional transitions of sarcopenia in older Chinese adults using CHARLS data [1]. Their letter raises several important methodological questions about the validity of estimating appendicular skeletal muscle mass (ASM) in large‐scale cohort studies [2]. We appreciate the opportunity to clarify our approach and discuss its alignment with international standards.

We fully acknowledge that dual‐energy X‐ray absorptiometry (DXA) is the reference standard for ASM measurement. However, in large, population‐based surveys such as CHARLS, DXA is not feasible due to logistical limitations and concerns regarding economic feasibility. Recognizing this, both the Asian Working Group for Sarcopenia (AWGS 2019) and the revised European Working Group on Sarcopenia in Older People (EWGSOP2) explicitly endorse the use of validated anthropometric equations when direct imaging is unavailable [3, 4]. Our study employed the widely used equation by Wen et al., developed and validated in a Chinese adult population, which has shown strong correlation with DXA‐measured ASM [5]. This equation has been widely adopted in longitudinal CHARLS studies examining outcomes ranging from mortality to cognitive decline.

A key concern raised was the age mismatch between the derivation cohort of the Wen equation and our elderly sample. While this is a valid point, the formula includes age, sex, height and weight, accounting for key covariates influencing muscle mass. More importantly, we applied an internal standardization strategy: defining low muscle mass based on the sex‐specific 20th percentile of ASM/height^2^ within the CHARLS population. This approach is well supported in epidemiological literature. In a population‐based study, Coin et al. demonstrated that applying the 20th percentile of ASM/height^2^ for an elderly reference population provided a more sensitive and appropriate method for identifying sarcopenia‐related risk than using absolute cut‐offs derived from younger populations [6]. The choice to use internal percentiles also addresses another concern: secular trends in anthropometric variables. Instead of applying fixed cut‐offs from external cohorts or historical reference data, internal thresholds inherently reflect the specific characteristics of the study population at that time. This improves classification accuracy, especially in populations where height and body composition may shift across generations.

Liu et al. also pointed out that more comprehensive anthropometric equations, such as those incorporating calf circumference, may improve ASM prediction. We agree that these newer models represent an important methodological advance. Indeed, a recently proposed calf‐inclusive anthropometric model for older Chinese adults demonstrated strong agreement with bioelectrical impedance analysis (BIA) measured ASM and holds promise for improved estimation accuracy [7]. However, calf circumference is not collected in CHARLS, precluding the application of such models in our analysis. Given these data constraints, we selected the Wen equation as the most feasible and validated option for this dataset.

Moreover, our sarcopenia classification followed the AWGS 2019 consensus, incorporating muscle strength and physical performance in addition to muscle mass. This multidimensional framework mitigates the influence of any single measurement and reflects functional status. Even if ASM estimation is imperfect, the inclusion of performance‐based criteria increases robustness and clinical relevance. Notably, the observation of sarcopenia reversibility in our study is consistent with findings from the 12‐year SNAC‐K cohort, which reported meaningful two‐way transitions [8].

In population‐based epidemiology, methodologic rigour must be balanced with practicality and consistency. Our approach satisfies current best practices: It is reproducible, transparent and context sensitive. We believe it represents a sound scientific method to advance understanding of sarcopenia in aging Asian populations, while remaining open to further refinement as future waves of CHARLS incorporate new anthropometric data.

## Conflicts of Interest

The authors declare no conflicts of interest.
